# Bridging Gender Gaps in Global Health: Insights from the Gender and Health Applied Learning Institute

**DOI:** 10.5334/aogh.4811

**Published:** 2025-09-03

**Authors:** Mary de Boer, Katherine Banchoff, Rosemary Morgan, Anna Kalbarczyk

**Affiliations:** 1 Department of International Health, Johns Hopkins Bloomberg School of Public Health, Baltimore, USA

**Keywords:** gender, applied learning, training, equity, evaluation

## Abstract

*Background:* Gender’s influence on health outcomes is well-documented, yet gaps in gender expertise persist within the global health workforce. Simultaneously, accessible and interactive gender training opportunities are limited. The Johns Hopkins Bloomberg School of Public Health Gender and Health Summer Institute (GHSI), launched in 2023, aims to address these gaps by advancing the gender integration and analysis skills of health professionals.

*Methods:* Using Stake’s Countenance Model for educational evaluations, we explored whether the Institute was meeting its objective of providing applied knowledge and experience of gender integration and analysis for health research, programs, and policy. The evaluation focused on intended and actual program outcomes. We examined proposal documents and held discussions with the GHSI team. All students receive pre-course surveys one week prior to each course. Post-course surveys focused on changes in knowledge, skills, and abilities and overall experience. Two focus-group discussions were held with students. Survey data were analyzed descriptively in R, and qualitative data were analyzed thematically.

*Results:* The pre-course survey received 137 unique responses; the post-course survey received 78 responses. Results indicate that the GHSI successfully met many of its intended goals, for example, by increasing participants’ knowledge and skills in gender analysis and integration as well as confidence in applying new skills. Learning was enhanced through creating safe and inclusive spaces. However, the courses’ short duration and lack of a sustained community of practice were identified as areas for improvement.

*Conclusion:* Findings underscore the importance of applied skills training and the need for ongoing support to fully equip professionals to address gender disparities in health. The GHSI’s virtual format also demonstrates a scalable, innovative approach other programs may consider. Finally, recommendations are provided for enhancing the GHSI and similar programs to better serve working professionals and foster a more equitable global health landscape.

## Introduction

The influence of gender on health outcomes has long been acknowledged in the fields of public health and international development [1]. Global frameworks such as the *World Health Organization’s Gender Policy* [2] and the *United Nations Sustainable Development Goals* (notably Goals 3 and 5) [3] emphasize the urgency of addressing gender disparities as a means to achieve both health equity and overall development.

Gender disparities in health are driven by unequal power relations, social norms, and differential access to resources and decision‑making. These disparities manifest across the health system: women may face delays in receiving care due to financial dependence or caregiving responsibilities [4, 5]; men may underutilize preventive services due to norms around masculinity [6, 7]; and gender‑diverse individuals often encounter stigma, discrimination, and legal barriers that limit access altogether [8–10]. These inequities not only result in worse health outcomes for marginalized groups but also create inefficiencies—overburdening emergency care, undermining disease prevention efforts, and eroding trust in health systems [11, 12]. When health systems do not address these structural inequities, they fail to deliver care that is effective, inclusive, and equitable for all [11].

This has led to frequent calls to do more and better work in analyzing and addressing gender disparities [13, 14], and in response, many global health agencies have increasingly prioritized gender over the last decade, despite the gaps in gender expertise in the workforce [15]. Training in gender and equity will be a critical step to filling these gaps and creating new paradigms in health. Closing gender data gaps and providing optimal patient care is central to treating women, men, and gender minority individuals safely and effectively across their lifespans. Gender and equity training, education, and learning help key actors identify and respond to inequities, as well as to allocate appropriate resources to implement equitable health systems. Tackling inequities in women’s, men’s, and gender minorities’ health rests on improved data collection and research alongside an acknowledgment of bias and willingness to confront it. Gender and equity‑specific training for a diverse workforce, from researchers to healthcare providers, is essential in tackling disparities in health and paving a more equitable path forward.

Although there is a clear need for and benefit to training in gender, gender analysis, and gender integration, accessible and interactive training opportunities remain limited. Launched in 2023, the Johns Hopkins Bloomberg School of Public Health Gender and Health Summer Institute (GHSI) advances the gender integration and analysis skills of health professionals. The Institute aims to reach working professionals who want to increase their experience with gender integration and analysis in health research, programs, and policy. Short‑term courses spanning three to five days are offered during an eight‑week term over the summer, with all classes taught exclusively on Zoom for four hours per day [16]. Courses can be taken for‑credit or not‑for‑credit at significantly reduced cost. This format increases accessibility and allows for global participation across multiple time zones. The reduced not‑for‑credit cost also enables greater participation of individuals based in low‑ and middle‑income country contexts. There are 18 courses within the GHSI, all of which focus on applied skills related to gender integration and analysis.

In 2024, we conducted an evaluation of the GHSI, to explore whether it was meeting its objective of providing learners with applied knowledge and experience of gender integration and analysis within health research, programs, and policy. In particular, this evaluation aimed to assess changes in gender‑related knowledge, confidence in application, and learner engagement within inclusive environments. While the results are beneficial for strengthening the GHSI, we feel there is benefit in sharing them publicly to help others develop similar programs with unique considerations for working professionals seeking to immediately practice and apply skills to improve gender and health outcomes in global health. This article reports the findings of the evaluation and provides recommendations for improving the instruction and organization of similar training programs.

## Methods

The evaluation focused on the 20 courses included in the 2024 GHSI. Courses spanned a range of topics, from policy‑related (Public Health Advocacy and Gender) to research‑based (Participatory System Modeling for Analysis of Gender Health Inequities) to professional development‑oriented (Essential Skills for Women’s Leadership in Global Health). All courses were delivered synchronously on Zoom and ran for four or five consecutive days.

The evaluation approach was predicated on Stake’s Countenance Model for educational evaluations [17] and was conceived of as a formative evaluation. We selected Stake’s Countenance Model because it is widely recognized for its comprehensive approach to educational evaluation, focusing on both the intended and actual outcomes of an educational program. It is highly context‑sensitive, emphasizing that the context of the program plays a significant role in the evaluation process; this is particularly beneficial for complex educational settings or diverse populations like those we targeted in the GHSI.

Stake’s Countenance Model assesses three areas: (1) antecedents, which signify a student’s pre‑existing aptitude, experience, and interest in the subject; (2) the educational transactions between the student and the teacher; and (3) the educational outcomes of that transaction in the form of the abilities, achievements, attitudes, and aspirations of the student. The educational outcomes need to be pre‑determined and aligned with appropriate instructor–student engagements (transactions), such as participatory approaches, applied learning, and inclusive structures, and be pitched at the appropriate level to align with students’ pre‑existing aptitudes, experiences, and interests in the subject (antecedents).

A formative evaluation, according to Stake’s framework, should compare the intended antecedents, transactions, and outcomes of a curriculum with what was actually observed by the evaluator. An educational curriculum is deemed congruent (or aligned) if the intended antecedents, transactions, and outcomes have come to pass. Similarly, it is deemed contingent (or connected) if the flow of antecedents, transactions, and outcomes are logical and supported by empirical evidence from multiple sources and previous evaluations, usually conducted in similar contexts. Establishing contingency requires evaluating whether one component logically depends on another.

**Figure 1:**
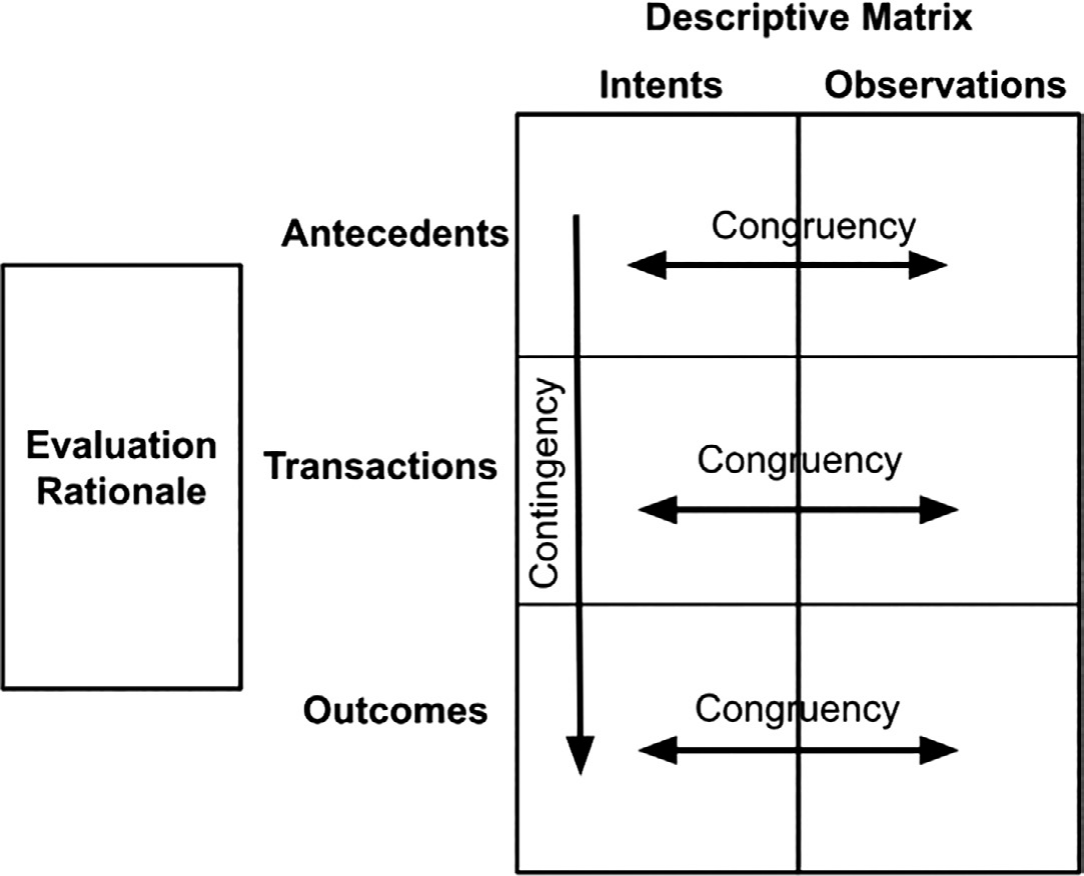
Stake’s Countenance Model for Evaluation—Descriptive (Formative) Evaluations.

**Figure 2:**
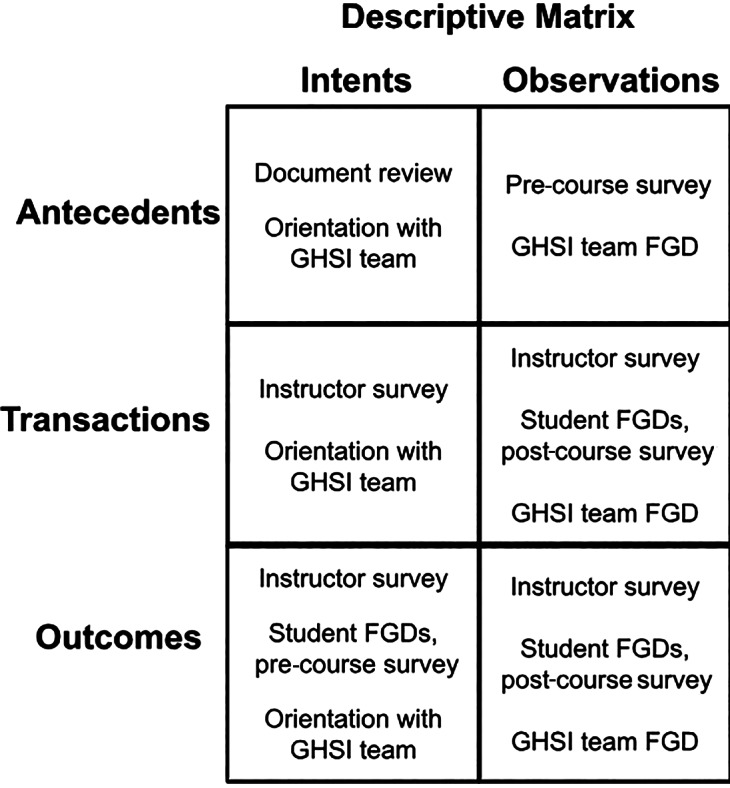
Data source for each component of the Stake matrix.

In this evaluation, we operationalized these comparisons by first extracting intended antecedents, transactions, and outcomes from planning and design documents, and then systematically comparing them to observed learner characteristics, reported course experiences, and self‑assessed outcomes from surveys and focus groups. Congruence was assessed by examining whether the intended elements occurred as expected, and contingency was evaluated by tracing whether learner characteristics logically aligned with course delivery and reported outcomes (Figures 1 and 2).

Specifically, to assess intended antecedents, the evaluator examined the proposal documents for the GHSI, reviewed grant and evaluation plan applications, and held discussions with the GHSI design team.

Observed antecedents were measured via a pre‑course survey, which was sent to all students registered in a GHSI course one week prior to the course’s launch. Students who did not complete the survey in the first week received a reminder three days before the course began. Eighteen students from two courses did not receive the reminder email, due to an internet connectivity issue. Each student was assigned a unique three‑digit student ID code, allowing the evaluation team to link their responses across multiple courses’ pre‑course surveys and between pre‑ and post‑course surveys.

The student pre‑course survey was 23 questions in length and included questions focused on the recruitment experience at GHSI (3 questions), questions on the scholarship experience—visible only for scholarship recipients (7 questions), course‑specific questions related to reasons for taking the course, pre‑existing knowledge, and confidence working on the topic (7 questions), and finally basic demographic questions (5 questions). The survey was hosted on Qualtrics.

To assess intended transactions and outcomes, the evaluators again examined the proposal documents, grant and evaluation applications, and GHSI design team feedback. Observed transactions and outcomes were measured via a post‑course student survey, sent after the end of all GHSI courses to students who had completed at least one course.

The base student post‑course survey was 23 questions in length, like the pre‑course survey, but could be as long as 49 questions for students who had enrolled in three courses (the maximum in the GHSI sample). Questions focused on changes in knowledge/skills/abilities following the course (13 questions), experience with the GHSI as a whole (5 questions), and a repeat of the demographic questions from the pre‑course survey (5 questions). This follow‑up survey was also hosted on Qualtrics. Individuals with partial/incomplete responses to the follow‑up survey were contacted individually and urged to complete the survey.

Additionally, two one‑hour‑long focus group discussions were conducted with five students (four women/one man, three international/two domestic) who indicated interest and availability.

Survey data were first analyzed descriptively to track the frequency of different responses using the Qualtrics software. Subsequently, to assess whether responses to a particular question varied significantly across response options, we sorted the data for each question into contingency tables in R and—due to the relatively small sample size for some categories—applied Fischer’s exact test for significance, using an alpha level of 0.05. All quantitative data were analyzed using R.

Qualitative responses were analyzed thematically using the six‑phase approach outlined by Braun and Clarke [18], with quotations organized in Google Sheets. Following an initial familiarization with the data, responses were grouped into primary and secondary thematic categories based on inductive patterns emerging from the data. These categories were iteratively reviewed, refined, and named, with exemplar quotes extracted to support interpretation. Significant differences in the frequency of responses across categories were also analyzed, using the same approach of contingency tables in R.

All participants provided informed written consent at the beginning of each survey; all focus group participants provided both written and oral consent before joining focus group discussions. The oral and written consent forms included assurances of confidentiality and voluntary participation. This evaluation was approved by the Johns Hopkins School of Public Health Institutional Review Board.

## Results

To assess congruency and contingency, the results are organized according to intended antecedents, observed antecedents, intended transactions and outcomes, and observed transactions and outcomes. By exploring intended and observed antecedents, transactions, and outcomes, we were able to examine the alignment between objectives and actual results.

### Participant demographics

The characteristics of the 137 unique survey participants are described in Table 1. The majority (60%) identified as women, with the two largest self‑identified racial and ethnic groups being Black (28%) and Asian (21%). Forty‑five percent of participants were in the 30–39‑year‑old age band, and the most common employers were civil society/non‑governmental organizations (37%).

**Table 1:** Survey participants.

DEMOGRAPHIC CATEGORY	PRE‑COURSE SURVEY (N = 137) N (%)	POST‑COURSE SURVEY (N = 78) N (%)
**Gender**		
*Women*	89 (59.7%)	48 (55.2%)
*Men*	25 (16.8%)	18 (20.7%)
*Cisgender*	26 (17.4%)	17 (19.5%)
*Genderqueer*	4 (2.7%)	1 (1.1%)
*Nonbinary*	3 (2.0%)	3 (3.4%)
*Transgender*	1 (0.7%)	–
*Not listed*	1 (0.7%)	–
**Ethnicity**		
*Native American*	2 (1.4%)	3 (3.7%)
*Asian*	31 (21.4%)	16 (19.8%)
*Black*	40 (27.6%)	28 (34.6%)
*Hispanic/Latino*	10 (6.9%)	7 (8.6%)
*Indigenous*	1 (0.7%)	–
*Middle Eastern*	6 (4.1%)	3 (3.7%)
*Pacific Islander*	1 (0.7%)	–
*South Asian*	15 (10.3%)	4 (4.9%)
*White*	27 (18.6%)	20 (24.7%)
*Not listed*	12 (8.2%)^1^	3 (3.7%)^2^
^1^ = African, Caribbean, Central American, Turkish, Brazilian, Prefer not to disclose ^2^ = African, Caribbean		
**Age band**		
*18–29*	37 (27.1%)	20 (25.6%)
*30–39*	61 (44.5%)	32 (41.0%)
*40‑49*	33 (24.1%)	20 (25.6%)
*50‑59*	2 (1.5%)	1 (1.3%)
*No answer*	4 (2.9%)	5 (6.4%)
**Employer**		
*Academia*	16 (11.7%)	13 (16.7%)
*Business*	4 (2.9%)	2 (2.6%)
*Civil society/non‑governmental organization*	50 (36.5%)	28 (35.9%)
*Consultant/ independent*	18 (13.1%)	12 (15.3%)
*Funder*	3 (2.2%)	1 (1.3%)
*Government*	15 (10.9%)	5 (6.4%)
*Student*	9 (6.7%)	4 (5.1%)
*Unemployed*	1 (0.1%)	1 (1.3%)
*Other*	17^3^ (12.4%)	7^4^ (9.0%)
*No answer*	4 (2.9%)	5 (6.4%)
^3^ = Community advocate, Hospital/clinical setting, International organization, multiple employers, UN ^4^ = Donor‑funded project, Hospital/clinical setting, International organization, UN		

The focus group discussions included five participants, the majority of whom were women (80%), employed by civil society organizations (60%), and based outside of the United States (80%); see Table 2.

**Table 2:** Focus group discussion participants.

DEMOGRAPHIC CATEGORY	PARTICIPANTS – N (%)
**Gender**	
*Women*	4 (80%)
*Men*	1 (20%)
**Employer**	
*Civil society/non‑governmental organization*	3 (60%)
*Student*	1 (20%)
*Government*	1 (20%)
**Geographic Location**	
*Outside the US*	4 (80%)
*USA*	1 (20%)

### Intended antecedents

Intended antecedents refer to the intended audience and their pre‑existing aptitude, experience, and interest in the subject. In reviewing the GHSI’s initial proposal and related documents, the intended target audience and related antecedents were met. From the beginning, the GHSI aimed to target working professionals, both currently in the field and those hoping to move into the field of gender and global public health. The design of the GHSI—short‑term and over the summer—was formulated with this audience in mind.

In addition, the GHSI team described in their proposal documents that they specifically hoped to target students with a pre‑existing interest in gender, namely students who recognized that “the implementation of equitable global health programs requires applied skills in gender and gender analysis in order for such programs to achieve an impact on health outcomes and equity.”

### Observed antecedents

Observed antecedents refer to the actual aptitudes, experiences, and interests of the students who enrolled in the GHSI. These were examined primarily in the student pre‑course survey; 236 pre‑course surveys were distributed to student participants, and 162 surveys were completed (a 69% response rate). Of these 162 complete surveys, 137 represented distinct students, as several students enrolled in multiple GHSI courses and completed the survey for each course.

Students completing the pre‑course survey indicated they chose to enroll in a GHSI course mainly due to the topic/subject matter of the course (87%) as well as to enhance their professional development (71%); 17% were attracted by a particular instructor or instructor(s). Most hoped to expand their theoretical knowledge of gender (35%), as well as to gain practical skills in gender analysis (33%). Those seeking practical skills shared goals, such as “How to do, step by step, practically, a gender assessment in a health program” (Student 119). Those seeking to expand their knowledge of gender were less specific in their aims, with many sharing goals such as “Learn about key gender frameworks and how to apply them to global women’s health” (Student 109) (See Table 3).

**Table 3:** Knowledge and skills students sought from the course.

THEME	WHAT KNOWLEDGE AND/OR SKILLS DO YOU HOPE TO GAIN AS A RESULT OF TAKING THIS COURSE? ILLUSTRATIVE QUOTES
**Application (33%)**	“How to do step by step, practically, a gender assessment in a health program”
**Knowledge (35%)**	“Learn about key gender frameworks and how to apply them to global women’s health”
**Research (12%)**	“How to apply sex and gender in the various stages of research”
**Measurement (8%)**	“Learn more about the ways we can measure sexual orientation, gender identity, and gender expression”
**Design (5%)**	“Developing health programs that account for gender equity”
**Policy (5%)**	“Gender‑based violence prevention and response and policy influencing and advocacy”
**Networking (2%)**	“I hope to interact with different professionals, make professional links and keep in conversation, learning from each other”

Similarly, while many articulated only general ideas about how they would apply course material (24%)—for example, by “bring[ing] a gender lens into my work” (Student 111)—a plurality shared very specific examples from their existing jobs (22%) and/or specific planned upcoming activities that they wished to prepare for (20%), such as contributing to policies, conducting analyses, or designing new programs (see Table 4).

**Table 4:** Students’ intended applications of the course content.

THEME	HOW DO YOU INTEND TO USE THE COURSE KNOWLEDGE IN YOUR CAREER? ILLUSTRATIVE QUOTATIONS
**Non‑specific job application (24%)**	“Bring a gender lens into my work”
**Specific aspects of existing job (22%)**	“I am currently working for a consortium working on disability inclusion—advocacy is a critical part of our program…. Women and girls with disabilities often face greater and more complex challenges in accessing health services and full enjoyment of their rights. I feel that a better understanding of gender‑transformative advocacy frameworks would be beneficial for how we design and deliver our program”
**Planned upcoming activity (20%)**	“To inform the development of a quality assessment on GBV services in LMICs”
**Research (17%)**	“I intend to use this course to develop my academic and research career in gender research for current and future work”
**Growth and mentorship (10%)**	“To promote the advancement of women, including myself, in public health”
**Preparation for new role (8%)**	“I hope to get a job in preventing disparities within women’s health, working in maternal care in a hospital setting”

Most students reported having either minimal (46%) or moderate (45%) knowledge of the material. Confidence levels were more mixed, with 22% expressing extreme confidence in applying the material, 32% moderate confidence, 36% reporting themselves to be somewhat confident, and only 11% indicating they were not confident.

### Intended transactions and outcomes

Intended transactions and outcomes refer to the intended processes, interactions, and activities that occur during implementation of the course. They also include educational outcomes resulting from the transactions in the form of students’ abilities, achievements, attitudes, and aspirations. The GHSI proposal and documents outline a specific vision for the type of learning transactions that the GHSI would offer. Instructors wanted to develop an inclusive learning environment within the institute, where materials that decentered instructors’ voices and lifted others’ experiences were curated. Examples that the GHSI team offered include working to engage students in drawing from and sharing their own experiences, developing reading lists that focus on minority group authors, and incorporating multiple ways of knowing beyond reading into the curriculum, for example, via the use of podcasts and/or videos in course materials. Specific tools were identified that could help across the curriculum, including offering translation and caption tools on Zoom to improve engagement among students who did not speak English as a first language.

In terms of outcomes, according to the document review, the GHSI aimed to offer students “skills and competencies in implementing gender analysis,” “strengthen their qualifications for obtaining gender specialist positions in government, non‑profits, and academia,” and “signal to potential employers that students have the skillset to work in this area.” The focus primarily on skills was prominent.

In the same way, in interviews with the GHSI team, team members placed special emphasis on applied skills, citing their desire to offer

“… concrete training […] on applied methods, including gender analysis, using data to promote gender equity and health, gender transformative interventions, conducting gender assessments, conducting secondary analysis of data to support gender analyses, gender and monitoring and evaluation, organizational change and leadership, creating an enabling environment for gender integration, advocacy and communication for gender equity, and gender‑based violence research methods.”

Ultimately, the GHSI hoped that students would be able to take their new skills back to their organizational environments and programming, augmenting the quality of gender analysis conducted at field level around the globe, and build a network or community of practice among practitioners who could support and enhance one another’s work.

### Observed transactions and outcomes

Observed transactions and outcomes refer to the observed processes, interactions, and activities that occurred during the course as well as the observed educational outcomes for the students. Students offered feedback on the educational transactions as well as educational outcomes via both the post‑course survey and—for interested students—subsequent FGD discussions. Post‑course survey response rates were lower than for the pre‑course survey: 176 post‑course surveys were sent out, accounting for dropouts from the initial registrants, and 78 complete responses were received (a 44% response rate). Seventy responses could be linked between the pre‑ and post‑course surveys.

According to the post‑course respondents, all felt that they had gained the knowledge and skills they expected from the course—24% saying “somewhat” and 76% saying “to a great extent.” There was no significant variation in knowledge gained based on participants’ reported initial knowledge of the topic from the pre‑course survey (Fischer’s exact test, p = 0.90).

In discussing the skills they gained, students highlighted that the content felt practical and applicable, citing that they liked “the practical approach of the course” (Student 146) and the “immediately applicable skills—as well as concepts that were both supportive and challenging” (Student 252). Others highlighted that they had benefited from the community and the broader experience of the course, as well—describing how the course had been a “a safe space,” due to “the community environment created over the 5 days” (Student 252). Participants also noted that they “gained valuable leadership skills and advocacy skills” in addition to “different perspectives on gender topics and women in leadership” (Student 237).

The post‑course survey also explored students’ opinions about the professional relevance of the GHSI courses. Participants reiterated the immediate practical value of the classes for them, indicating that “my job is currently doing this type of analysis” (Student 220) and that “I have already applied things from the course to our work” (Student 101). Several also noted that they had shared the course content with colleagues. Others emphasized the utility of the course in supporting career transitions or adjusting career trajectories (Student 256, Student 277).

**Figure 3:**
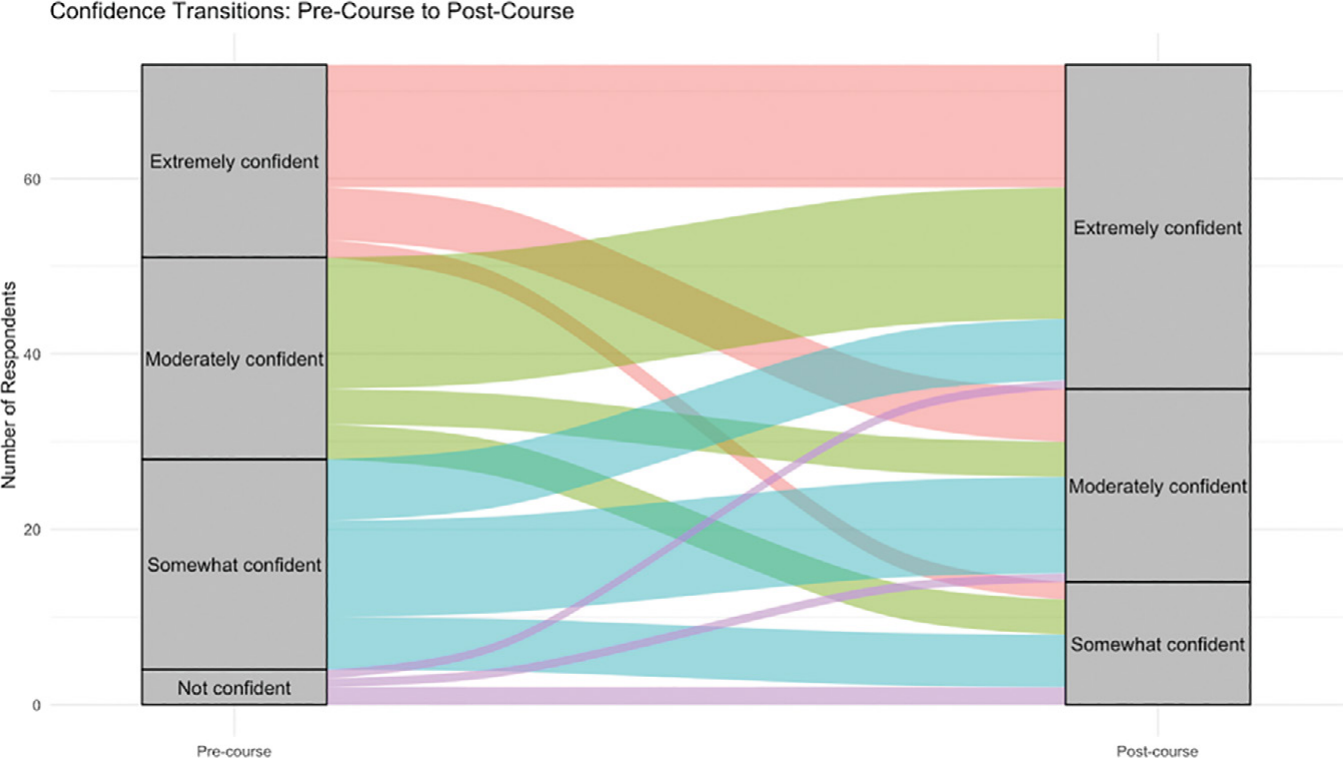
Trends in confidence between pre‑ and post‑course assessments.

In examining pre‑ and post‑course survey data, trends were most notable when respondents rated their confidence in applying skills they had learned in the GHSI courses. Students tended to move from having lower to higher levels of confidence following course delivery: for example, of the 22 students who expressed moderate confidence in the pre‑course survey, 14 had moved to being extremely confident at the time of the post‑course survey (67%). Similarly, of the 25 students who rated themselves as somewhat confident in applying the course content during the pre‑course survey, 11 had moved up to moderately confident (44%) and 7 to extremely confident (28%) by the post‑course survey. These trends were marginally significant (Fischer’s exact test, p = 0.062; Figure 3).

In examining how trends in confidence differed based upon demographic characteristics, we saw that men had higher confidence in their skills overall, and this confidence tended to increase between pre‑ and post‑course surveys. Women, whites, and Asians were more likely to say that they were not confident at the outset, while blacks as a group had the highest levels of confidence in applying GHSI materials at the completion of the course (Table 5).

**Table 5:** Trends in student confidence by demographic characteristics—proportion of students indicating the given confidence level in applying course material at pre‑ and post‑course timepoints.

	PRE‑COURSE				POST‑COURSE			
	EXTREMELY CONFIDENT	MODERATELY CONFIDENT	SOMEWHAT CONFIDENT	NOT CONFIDENT	EXTREMELY	MODERATELY	SOMEWHAT	NOT CONFIDENT
**Primary Race/Ethnicity**								
A racial/ethnic identity not listed here (n = 2)	0.50	0.50	0.0	0.0	0.50	0.00	0.50	0
Asian (n = 14)	0.36	0.07	0.43	0.14	0.43	0.43	0.14	0
Black (n = 26)	0.38	0.31	0.27	0.04	0.65	0.19	0.15	0
Hispanic/Latino/x (n = 4)	0.00	0.75	0.25	0.00	0.25	0.25	0.50	0
Middle Eastern (n = 2)	0.5	0.00	0.5	0.00	0.00	1.00	0.00	0
South Asian (n = 3)	0.00	1.00	0.00	0.00	0.33	0.67	0.00	0
White (n = 13)	0.23	0.15	0.54	0.08	0.38	0.38	0.23	0
**Primary Gender Identity**								
Cisgender (n = 6)	0.33	0.50	0.17	0.00	0.33	0.50	0.17	0
Genderqueer (n = 1)	1.00	0.00	0.00	0.00	1.00	0.00	0.00	0
Man (n = 17)	0.38	0.38	0.25	0.00	0.69	0.31	0.00	0
Nonbinary (n = 2)	0.00	0.50	0.50	0.00	0.00	1.00	0.00	0
Woman (n = 39)	0.28	0.21	0.41	0.10	0.44	0.28	0.28	0

When asked to explain their confidence levels, students were enthusiastic about the GHSI, describing how the courses served as a strong preparation for real‑world applications. One student shared: “The course offered numerous practical applications and case studies that allowed me to apply the concepts in real‑life scenarios” (Student 104), while another stated, “I feel extremely confident because we were not just taught but supplied with resources” (Student 226). Students reflected on how their confidence had expanded from pre‑ to post‑course as a result of this targeted skills‑building, with one student noting, “I already had some foundational knowledge, and the course helped me to outline the steps [of gender analysis] more definitively,” noting she was now “extremely confident” to apply the materials (Student 141).

However, many students also felt more practice was needed, describing that while they felt “well‑equipped to start applying what I’ve learned …I still need more hands‑on experience and exposure to diverse contexts to fully refine and adapt these skills” (Student 125). Similarly, another student pointed out: “Implementing knowledge comes with practice and experience, it cannot be instantaneous after attending classes” (Student 129).

Reflecting this trend, when students were asked to assess the structure of the GHSI as a whole, they were strongly positive about the skills‑building aspects of GHSI courses, with 93% agreeing strongly with the statement that “GHSI offers courses that build skills in various aspects of gender,” and 68% strongly agreeing that “GHSI’s courses include applied methods and are grounded in real world challenges.”

Conversely, many students underscored that the depth and length of the institute’s courses were drawbacks. One student shared that “Since the course was short, most topics were introduced rather than explored in depth,” (Student 149), while another offered: “I don’t think the course was in‑depth enough to bring substantial value, but it was a good starting point” (Student 200).

Others commented on the inherent tension of online learning and professional development courses: “The course was done over a 4‑day period so it was a lot of information in a short space of time considering I also had to work,” (Student 232) with another sharing “In some ways I wish it were longer, but practically speaking it was also better to keep it short” (Student 141).

Finally, in discussing the openness of the learning environment and approaches to co‑learning, students cited the “collaborative learning process” they had experienced (Student 220) and shared how helpful the conversations and resources had been that were brought into the classroom by fellow students (Student 165). They also discussed the diversity of the class (Student 197), the use of alternative learning methods like videos and guest speakers (Student 228, Student 232), and that the class was a “safe space to discuss the problems we face as women in leadership” (Student 253). In discussing the personal impact of what they had gained from the GHSI, one student noted that “some of the discussions opened windows into my perspective, which will for sure stay with me in my professional life” (Student 211).

Students critiqued that this collaborative learning environment did not persist after the end of the course. This was framed in multiple ways in the post‑course survey: some requested more opportunities for networking before, during, and after the courses (Student 153, Student 307), others requested “additional resources or follow‑up sessions for ongoing support and continued learning” (Student 271), while still others asked for “more opportunities for one‑on‑one mentorship or networking sessions with professionals in the field” (Student 104). Overall, there was a desire for “continued learning … as a cohort from each other” (Student 268).

Table 6 outlines the ways in which congruence was achieved (or not) between intentions and effects across antecedents, transactions, and outcomes.

**Table 6:** Congruence between intentions and observed effects.

	INTENTS	OBSERVATIONS
**Antecedents**	Professionals working in the field Those interested in doing more work in gender People being asked to do gender‑sensitive programming and gender analyses but do not know how	Students taking courses were primarily motivated by professional development and topic/subject matter Two top interests were in building knowledge and gaining applied skills Some students had specific upcoming activities they wanted support on
**Transactions**	Focusing on applied learning Creating learning environments that are flexible, convenient, challenging and that empower adult learners GHSI is a safe space to explore difficult identity‑challenging topics	Applied skills were well transmitted and understood The learning environment was enjoyable and safe Challenges with duration/depth
**Outcomes**	GHSI is a one‑stop shop for gender and health professionals Establishment of an ongoing community of practice	Gains in knowledge/skills High professional relevance Increased confidence, although lower than desired in terms of independent application Lack of community after the course completion

## Discussion

Evaluation results showed that the GHSI successfully met many of its intended goals. The GHSI aimed to target working professionals in search of applied skills, and indeed the pre‑course survey indicated that these were largely the type of students who enrolled in the GHSI courses—students came primarily from the NGO sector, many had a moderate familiarity with the topics of their courses, and a large proportion were taking the course in order to advance their professional development.

In evaluating the complementarity between intended and observed educational transactions and outcomes, the data also support a high degree of congruence. The GHSI targeted the transmission of applied skills, and students frequently shared positive feedback on the professional impact and relevance of the coursework. Notably, students who began the course with a specific project in mind, either one that was ongoing or shortly pending, expressed the highest satisfaction with the courses. These students may best represent the target population of the Institute: working professionals with pre‑existing engagement in the field.

These students’ satisfaction is largely supported by the literature on adult learning, which indicates that adults learn best when instruction draws from the learner’s own experiences, is immediately useful, and engages the learner in applying the material [19–21]. It appears that the GHSI, in adopting these approaches, has been able to successfully increase the skills and knowledge of many of its students.

While the focus and interest in gaining applied professional skills was high, the most frequently stated motivation to attend the GHSI was to gain theoretical or more general knowledge on gender, which contrasted with the institute’s goal of focusing on applied skills. Many courses assumed prior knowledge, with some providing introductory background reading or brief recordings or introductory sessions introducing gender. As a large proportion of students expressed limited knowledge about the topic of gender, explicitly meeting this expectation will be important for future iterations of the institute.

Despite the applied skills focus, the duration of GHSI courses felt brief for many students, and they cited a resultant lack of confidence in applying course materials independently. While confidence increased from the pre‑course surveys, students underscored that more practice was necessary to feel fully confident. This may reflect a common learning trajectory seen across all age groups: learners often begin unaware of the depth of a topic and feel confident in their assumptions. However, as they engage more deeply, they become increasingly aware of the subject’s complexity and may feel less confident—even as their knowledge and skills improve [22, 23]. Continued growth, including experiential learning and support from colleagues, is often essential for building fluency and ease in applying new material [24–26].

The lack of opportunities for continued growth speaks to an unrealized aspect of the GHSI—the goal of establishing a community of practice to support the GHSI participants and facilitate the sharing of resources, opportunities, and feedback. Virtual communities of practice (VCoPs) have been increasingly studied in recent years, and several aspects of the GHSI approach may make fostering a VCoP especially challenging. First, VCoPs that are established rather than spontaneously emerging can struggle to foster and sustain interest and collaboration [27]. Similarly, the need to cross multiple organizational, geographic, and cultural boundaries can further affect trust and reduce the sharing and vibrancy of VCoPs [28]. Successful VCoPs have all required purposeful nurturing [29], with many possessing very involved and innovative leaders and/or leadership groups, something that is currently missing from the GHSI community [30]. Finally, the relevance of VCoPs to their members’ daily work has been a crucial predictor of member satisfaction and longer‑term success [31, 32]. Ensuring a successful VCoP may require greater focus and investment on behalf of the GHSI administrative team, including sustained, active engagement with alumni members to ensure their needs are being met.

However, the virtual nature of the GHSI is simultaneously one of its greatest strengths. Online, synchronous, multi‑regional learning platforms can create opportunities for intercultural connection, increasing participants’ access to diverse perspectives, growing their peer networks and increasing their access to experts in the field [34, 35]; these types of experiences were highlighted repeatedly by survey respondents and focus group interviewees. While some learners were frustrated with the length of the online interactions, research indicates that the length is usually not the key determinant of success for professional development opportunities. Instead, the degree of applicability of the content, learner engagement with materials, and quality of delivery appear to determine uptake [36, 37]—areas where the GHSI excelled.

Finally, the GHSI aimed to create a safe, inclusive space where learners could safely examine issues of identity and equity; this was underscored in the post‑course survey and in focus group discussions with student participants. The institute was intentional in its use of universal design principles to enhance engagement with virtual learners and create safe and inclusive spaces [33]. For example, courses highlighted diverse perspectives by inviting guest speakers, using open‑access materials from an array of sources including non‑academic sources, and targeted the inclusion of non‑Western voices, ways of thinking, knowing, and doing. Synchronous and asynchronous learning modules were available; all synchronous modules used automatic live transcription and translated Zoom captions into 39 languages – this was foundational to effectively reaching a truly global audience. We also used collaborative online platforms for participants, instructors, and institute‑affiliated staff, which enabled us to integrate a variety of co‑learning approaches. This included novel online participation tools, flipped classrooms, and interactive activities that encouraged collaboration. Overall, the environment appears to have facilitated both professional and personal learning and a desire to continue connections.

Going forward, there are multiple opportunities for growth within the GHSI, some of which are already being realized. Alumni mentorship opportunities are being offered via platforms such as LinkedIn and Slack, and the institute has been approached with opportunities to develop tailored curricula for specific settings and organizations. The institute is also considering—as has been done by other similar programs—extending the duration while keeping the total number of contact hours the same. This approach, too, will need to be evaluated.

## Strengths and Limitations

A potential limitation of this evaluation was the 44% response attrition between the pre‑ and post‑course surveys. While this response rate is low, it falls within the typical range (40%–60%) for voluntary post‑program evaluations and is consistent with published benchmarks for survey research in educational and organizational contexts [38, 39]. Non‑response bias has the potential to select a sample population that differs in fundamental but difficult to assess ways from the target population [40]; however, higher response rates do not always result in more representative data [41]. We can only speculate that our sample includes those who were most motivated to share their opinions—positive or negative—about the GHSI.

Strengths of this evaluation include the fact that this evaluation is ongoing—respondents will be contacted again after one year to determine whether gains were maintained, offering the possibility of further insights into how to improve and strengthen gender training. The online, multi‑modal learning environment that allowed for broad geographic and socio‑demographic participation is also a strength of the program, reflected in the survey respondents. Finally, the inclusive pedagogical approach, building on fundamental principles of adult learning, liberatory education and postcolonial theory, demonstrates how the GHSI is striving to embody the values it promotes.

## Conclusions

Evaluation results showed that the GHSI successfully met many of its intended goals by targeting working professionals with pre‑existing engagement in the field and offering applied skills in gender integration and analysis. Students reported gaining significant knowledge and skills from the courses. The practical orientation of the courses was particularly appreciated, with students noting the immediate applicability of the content to their work. However, the evaluation also identified areas for improvement, such as the need for more practice to build confidence in applying new knowledge and skills independently and the need to establish a virtual community of practice. For some, a week‑long, online classroom experience was brief and intense; however, this approach was appreciated by others who needed to take the course alongside their professional responsibilities. The GHSI has made significant strides in advancing gender integration and analysis skills among health professionals. However, there remains a need for continued support and opportunities for practice to ensure sustained confidence and application of these skills.

Indeed, the GHSI may prove a helpful model for other types of training opportunities in the global health space. Gender is a topic that is evolving and one on which many practitioners still require training. The GHSI model of professional development—online and brief but also focused and practical—is ideal for similar emerging areas of interest and focus for the public health development community, spanning ministries of health, non‑governmental organizations, and academic research centers. The GHSI format and content are well adapted for this purpose; others may wish to adopt similar approaches going forward.
